# Measuring spatial accessibility of residents’ medical treatment under hierarchical diagnosis and treatment system: Multi-scenario simulation in China

**DOI:** 10.1371/journal.pone.0282713

**Published:** 2023-04-10

**Authors:** Zhongyu Wei, Jianjun Bai

**Affiliations:** School of Geography and Tourism, Shaanxi Normal University, Xi’an, China; Springer Nature, UNITED STATES

## Abstract

In order to improve the operational efficiency of medical institutions and build a more complete and efficient medical system, the Chinese government is vigorously promoting the reform of hierarchical diagnosis and treatment. We constructed a multi-factor composite selection weight to characterize the residents’ medical treatment behavior in the context of hierarchical diagnosis and treatment. By combining the weight with the two-step floating catchment area method, we analyzed the spatial variation characteristics of residents’ accessibility to medical care under different scenarios. Results show that the referral rate between medical institutions increases gradually along with the occurrence of public health events. When there is a major public health event, the proportion of the population transferred from the primary medical institutions to the county hospitals and the county hospitals to the municipal hospitals exceeded 65%. In three scenarios, the spatial pattern of accessibility shows obvious consistency and local differences. Among the three-tier medical institutions in China, the service capacity of county hospitals is poor, and the contribution rate of accessibility is less than 20%. The results clearly show the spatial differences in the accessibility of Chinese residents in different scenarios and the impact of public health events on accessibility. This research can provide a reference for the layout optimization of medical resources in the future.

## Introduction

As an important part of the public service system and the material carrier of medical and health services, the medical and health facilities play a fundamental supporting role in maintaining the health of residents [1–3]. With the rapid development of Chinese economy, more and more financial funds have been invested in the field of medical and health services [4]. From 2010 to 2020, the number of medical institutions in China continued to grow, and the types of medical institutions were constantly enriched. However, the improvements in the physical conditions of medical and health facilities cannot fundamentally boost the operational efficiency of health systems [5]. The hierarchical diagnosis and treatment system based on referral, which strictly triages patients according to the function of medical institutions at different levels, has obvious advantages in improving the operational efficiency of medical institutions and has been widely used. For example, the developed countries such as the United Kingdom, the United States and Japan have formed an efficient hierarchical diagnosis and treatment system [6]. In 2009, a new round of medical policy reform was introduced, and the hierarchical diagnosis and treatment system was proposed to be established in China for the first time [7]. In 2015, the State Council of the PRC issued the "Guiding Opinions on Promoting the Construction of the Hierarchical Diagnosis and Treatment System" to further clarify the construction direction of the hierarchical diagnosis and treatment system [8]. In the past 23 years, there have been some achievements in establishing the hierarchical diagnosis and treatment system in China. However, the initial goal of the reform has not yet been achieved, and the China’s medical situation has still been in a mixed treatment model for a long time. The hierarchical diagnosis and treatment system can improve the operational efficiency of medical facilities, but how to determine the referral rate among hospitals at each tier and the relationship between referral rate and accessibility needs to be further studied.

The concept of accessibility is from transportation geography, which is used to represent the spatial interaction of opportunities and has rich connotations and extensions [9]. Accessibility is not only used to assess how easily residents can obtain resources, but also plays an important role in policy making [10]. Therefore, accessibility is widely used in the evaluation of public facilities. For example, the ease of access to public resources for residents can be analyzed by calculating accessibility [11–13]. The combination of accessibility, Gini coefficient and Lorenz curve can be used to assess the equity of spatial distribution of public resources [14, 15]. The accessibility is also used as the evaluation index for the siting optimization analysis of public resources [16, 17].

In order to improve the accuracy of accessibility evaluation and meet the diversified needs, scholars have been working on the evaluation methods of accessibility. In previous studies, researchers used the distance and ratio methods to calculate accessibility due to limitations on data and analysis tools. For instance: Talen and Anselin [18] used the shortest travel distance to measure the accessibility of public playgrounds. Makuc et al. [19] divided the study area into 802 service areas and measured the accessibility of medical services by calculating the ratio of physicians and hospital beds per 100,000 population in the service area. This method is easy to calculate and understand, but ignores the spatial interaction between people and public facilities [20]. The two-step floating catchment area method, which was proposed by Luo&wang [21] in 2003, can take into account the service capacity of the supply point and the needs of people, and has been commonly used. However, as for the earlier two-step floating catchment area method, the accessibility of settlements within the threshold range is the same, but the settlements outside the threshold range are completely unreachable. This simple dichotomy has been questioned by some scholars [22]. To solve this problem, researchers extended the form of two-step floating catchment area method by introducing different types of decay functions, commonly used are Enhanced 2SFCA method [23, 24], Gravity2SFCA method [25], Gaussian 2SFCA method [26, 27], and kernel density 2SFCA method [28]. However, the two-step floating catchment area method suffers the limitation that ignoring the existence of competitive effects between facilities, especially when multiple facilities within the search radius of a demand point. Thus, Wan et al. [29] proposed a three-step floating catchment area method, which adds one step based on the two-step moving search method: calculating the selection weights of demand points to measure the competitive effects of multiple facilities within the threshold range of demand points, further enriching the research method of accessibility.

Subsequent researches in accessibility have been enriched on study scale and data precision. In terms of study scale, the accessibility at different scales showed different characteristics. In recent years, the study scale has gradually been improved from the statistical unit of the census (i.e., provinces, cities, counties, and towns in China) to communities or residential areas. For example, Rong et al. [25] studied the accessibility of Greenland Park in Zhengzhou from the scale of the residential areas. In order to reduce the influence of edge effects, scholars used the spatial grid data on the accessibility studies. Such as: Zhang et al. [30] divided the study area into 50m*50m grids to assess the accessibility of green spaces at a finer scale. The improvement of data accuracy is mainly based on population data and resident travel cost data. Along with the refinement of the research unit, the demographic data at the corresponding scale cannot be obtained. Thus, in the previous studies, the large-scale population data was distributed equally to small-scale areas based on the assumption that the population is equally distributed within statistical units [31]. With the abundance of multi-source geospatial data, it provides the possibility to obtain more granular demographic data. Chen et al. [32] used the floor height and the area of the building to estimate the number of people that the building can accommodate. The resident travel cost data refers to the cost of time and distance spent by residents from the starting point to the destination. The two-step floating catchment area method usually uses the Euclidean Distance or the transportation network distance to calculate travel cost data [33]. It has been observed that the traditional method ignored the dynamic changes in traffic conditions and the diversification of traffic forms, and it is difficult to accurately estimate the cost of transportation under different modes of travel. Then the traffic big data appeared and was widely applied. Some scholars used the map navigation services provided by online map service providers to obtain residents travel cost data under different travel modes. The traffic big data considered various factors such as traffic conditions, roads, and weather comprehensively, and have higher accuracy [34].

Under the framework of the hierarchical diagnosis and treatment system, we used accessibility as an indicator to measure the convenience of residents to medical services. However, given the form of hierarchical diagnosis and treatment, researchers assessed residents’ medical accessibility by constructing the multi-stage 2SFCA method. There are relatively mature researches on the accessibility of medical treatment with rich results. But there is few research conducted on the accessibility of residents to medical treatment under the hierarchical diagnosis and treatment system. And there are limitations in the existing research. First, there is no general agreement on the process of hierarchical diagnosis and treatment, which cause differences in the results of accessibility. Nie & Feng [35] divided medical institutions into low-level medical facilities and high-level medical facilities. Under the hierarchical diagnosis and treatment system, there are two stages when residents seeking medical treatment: medical treatment in low-level medical institutions and referral to high-level medical institutions. Chen Lu et al. [36] built a three-tier referral system for medical institutions: Patients first go to the primary medical institutions for treatment. Then the primary medical institutions offer step-by- step referral options according to patient condition, and even can request a referral to the highest level of medical institutions directly. Thus, a four-stage referral procedure is formed. Second, some studies calculated the proposed referral rates for hierarchical care systems based on the accessibility. For example, Nan Yang et al. [37] believed that when the referral rate is 1.24%, the accessibility of residents to medical service is the best. Zhong et al. [38] thought that it is the most convenient for residents to obtain medical resources when the referral rate is around 60%. Both studies were based on setting multiple referral rates and calculating accessibility to derive optimal referral rate. However, the optimal referral rates derived by different researchers differed widely. It suggests that referral rates determined by the accessibility are not representative and difficult to used for other study cases directly. In addition, only using the indicator of accessibility to determine the referral rate does not consider the utilization efficiency of medical resources at different levels, which is inconsistent with the original intention of the hierarchical diagnosis and treatment system to improve the utilization efficiency of hospitals.

This paper simulated the referral rate of hospitals from the perspective of hospital effective utilization, and analyzed the geographical distribution characteristics of accessibility of medical service for residents. At the same time, we believe that the referral rate of multi-tier hospitals is not fixed, which varies in different medical scenarios. The contributions of this paper are as follows:

1. The multi-stage two-step floating catchment area method that satisfied the hierarchical diagnosis and treatment system was proposed. The multi-factor comprehensive selection weight of hospital was added to the 2SFCA method to represent resident choice of hospitals.
2. We set up three scenarios for medical and health services: business-as-usual scenario, general public health case scenario, and major public health case scenario. And we analyzed the referral rates among medical institutions at different levels and the differences in the spatial distribution of accessibility in different scenarios.
3. The contribution rate of multi-tier hospitals to accessibility was further simulated with the aim to provide targeted opinions and suggestions for each province in China.

## The hierarchical diagnosis and treatment system & data

### The hierarchical diagnosis and treatment system

The hierarchical diagnosis and treatment system is an important measure to improve the efficiency of medical care services, rationally use medical resources, and save medical expenses. In 2015, China vigorously promoted the construction of graded diagnosis and treatment system including initial treatment in the primary institutions, the bi-directional referrals, the acute and chronic diseases treated separately, establishing cooperation between the upper-level and lower-level hospitals. The main purpose of the reform of hierarchical diagnosis and treatment is that medical institutions at all levels perform their own duties and the functional orientation of their own medical services can be clarified. Among them, the primary institutions are mainly responsible for the diagnosis and treatment of common and frequently-occurring diseases within their jurisdiction, and refer patients who are beyond their own scope of diagnosis and treatment. The county-level hospitals are in charge of the diagnosis and treatment for patients referred by the primary institutions and rescue severe patients. When facing with the patients in serious and critical condition, it is necessary to carry out upward referral. As the medical institution with the highest medical level, the municipal hospitals are mainly responsible for the diagnosis and treatment of difficult and severe diseases referred by the primary institutions and the county-level hospitals. Obviously, medical institutions do not work in isolation. There is support assistance between the upper-level and lower-level hospitals to continuously improve the medical service level of the lower-level hospitals.

### Data collection

#### Population data

We took the townships (streets) as the smallest research unit. In China, the township is the lowest census unit, but the population data of townships published by some provinces is missing. Thus, the township population data used in this paper is from the statistics of the township boundary based on the national kilometer grid population data. Among them, the national kilometer grid population data comes from the China Resource and Environment Science and Data Center in 2015. The population data were used as indicators representing medical needs in this study.

#### Hospital data

The public medical institutions play a decisive role in providing medical services. The hierarchical diagnosis and treatment system is mainly promoted in the public hospitals. Therefore, this paper took the public medical institutions of China as the research object, and divided the hospitals into primary medical institutions, county hospitals, and municipal hospitals according to the administrative affiliation. The point data of hospitals in China is from Baidu POI, which the attributes include the name, latitude and longitude coordinates of hospital. The data from Baidu POI only contains basic attribute information. We obtained attribute information such as the hospital grade and the number of beds from the official websites of the county and municipal hospitals. The number of beds in the primary medical institutions cannot be obtained, so we used the per capita beds of the primary medical institutions in each province as estimated value.

#### Travel path data

The road data from OpenStreetMap has become a commonly used road network database because of its fast update speed and complete data. The transportation network dataset in our research was established based on the road data from OpenStreetMap to calculate the distance cost between the starting point and the destination. The transportation network dataset in our research is more accurate compared with the results using the Euclidean distance of two points.

## Research methods

### Incorporating the multi-factor comprehensive selection weight into 2SFCA

Step 1: Calculating the supply and demand ratio

When calculating the supply-demand ratio, the traditional two-step mobile search method takes the supply point as the center of the circle, and delineates the corresponding service scope for each hospital according to the time or distance threshold to count the population within the threshold [39]. In this paper, the service scope of the hospital is determined by the administrative division. We believe that the residents within the corresponding service scope of the supply aspect have equal opportunities to obtain medical services at same levels. Therefore, when calculating the supply-demand ratio, this paper regarded medical institutions of the same level in the same district as a whole. And then sum the number of beds was divided by the population in the corresponding administrative division. The supply-demand ratio is calculated as follows:

$$R_{j}=\frac{S_{j}}{\sum_{i\in \left(d_{ij}\leq d_{0}\right)}P_{i}}$$ (1)

where *S*_*j*_ is the services capacity of the hospitals, represented by the number of beds; *P*_*i*_ is the population in region *i*.*d*_*ij*_ is the distance between *i* and *j*. *d*_0_ represent distance threshold.

Step 2: Constructing multi-factor comprehensive selection weights

**Fig 1:**
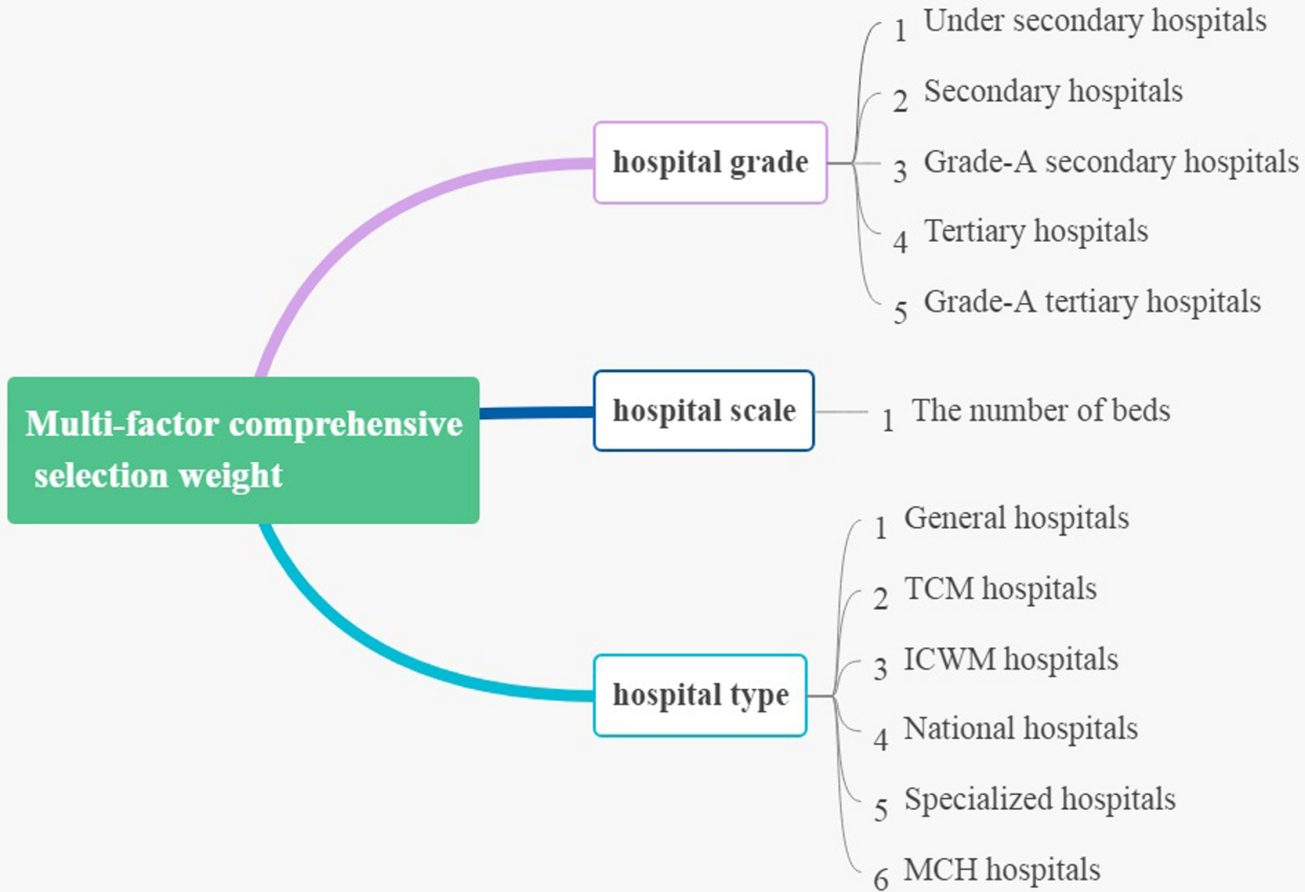
Constructed multi-factor comprehensive selection weights.

We proposed the multi-factor comprehensive selection weight (Fig 1), which considered different preferences and demands for medical treatment of residents and can rationally distribute residents to different types of hospitals. The scientificity and rationality of factor selection directly affect the selection results. According to the availability and measurability of data, we chose the hospital grade (X1), the hospital scale (X2), and the hospital type (X3) to construct the comprehensive selection weights. Among them, the hospital scale was expressed by the number of beds, which is a quantitative index and can be measured directly. The hospital grade and the hospital type are qualitative indicators, which need to be quantified and data standardized. Based on relevant literatures, this paper set the grade coefficient of the tertiary hospital to be twice that of the second-grade hospital, and the grade-A hospitals increased by 0.5 [40] (see in Table 1). The quantitative standard of the hospital grade coefficient is shown in the following table. The quantification standard of the hospital type is based on the number of patients diagnosed in medical and health institutions according to the 2019 National Health Statistical Yearbook [41]. The selection probability of a certain type hospitals can be obtained by dividing the annual diagnosis and treatment population of a certain type hospital by the total diagnosis and treatment population of all hospitals. The selection probability of each type hospital is shown in the Table 2:

$$T_{ij}{=\text{X}}_{1}{}^{*}+{\text{X}}_{2}{}^{*}+{\text{X}}_{3}{}^{}$$ (2)

$$G_{ij}=\frac{T_{ij}}{\sum_{k\in \left(d_{ik}\leq d_{0}\right)}T_{ik}}$$ (3)

Where *G*_*ij*_ is the multi-factor comprehensive selection weight between location *i* and medical facility *j*; *T*_*ij*_ and *T*_*ik*_ represent the weight from location *i* to the medical facility *j* or *k*; ${\text{X}}_{1}{}^{*}、{\text{X}}_{2}{}^{*}$ represent the standardized values of the hospital grade and the hospital size; X_3_ is the selection weight of the hospital type.

Step 3: Calculating accessibility

$$A_{i}=\sum_{k\in \left(d_{ik}\leq d_{0}\right)}R_{k}f\left(d_{ik}\right)G_{ij}$$ (4)

where *A*_*i*_ the accessibility of the resident *i*, *f* (*d*_*ik*_) gravity attenuation function.

**Table 1:** Hospital grade assignment.

Hospital grade	Under secondary hospitals	Secondary hospitals	Grade-A secondary hospitals	Tertiary hospitals	Grade-A tertiary hospitals
Grade assignment	0.5	1	1.5	2	2.5

**Table 2:** The selection probability of each type hospital.

Hospital type	General hospitals	TCM hospitals	ICWM hospitals	National hospitals	Specialized hospitals	MCH hospitals
Selection weight	0.6694	0.1418	0.0176	0.0036	0.0919	0.0756

Note: TCM hospital (Traditional Chinese Medicine hospital); ICWM hospital (Integrated traditional Chinese and Western Medicine Hospital); MCH (Maternity & Child Healthcare Hospital)

### The multi-stage 2SFCA for hierarchical diagnosis and treatment system

In the hierarchical diagnosis and treatment system, residents can obtain medical services from primary medical institutions, county hospitals and municipal hospitals. Therefore, the accessibility of residents to medical resources based on the hierarchical diagnosis and treatment system includes four categories, which can be expressed as:

$$A_{i}=A_{i}^{p}+A_{i}^{c}+A_{i}^{m1}+A_{i}^{m2}$$ (5)

The first stage is from residential areas to primary medical institutions. According to the hierarchical diagnosis and treatment system, residents need to go to the primary medical institutions for treatment, and primary medical institutions decide whether refer patient to the superior hospital according to the patient’s condition. At this stage, the demand point is the residents, and the supply point is the primary medical institution. The calculation formula is as follows:

$$R_{p}=\frac{S_{p}}{\sum_{k\in \left(d_{kp}\leq d_{0}\right)}P_{k}}$$ (6)

$$A_{i}{}^{p}=\sum_{p\in \left(d_{ip}\leq d_{0}\right)}R_{p}f\left(d_{ip}\right)G_{ip}$$ (7)

where *S*_*p*_ is the capacity of the primary medical institution *p*; *P*_*k*_ is the population served by the primary medical institution. The hierarchical diagnosis and treatment requires residents to the primary medical institution for initial diagnosis, so it is the entire population of the settlement *k*. *f* (*d*_*ip*_) is the Gravity-type distance decay function; *d*_*ip*_ is the cost of the distance from the settlement to the primary medical institution; *d*_0_ represents the distance threshold. This paper used the administrative units to delineate the service scope of medical facilities (the service scope of the primary medical institution is the township where they are located, the county hospital covers the entire county, and the municipal hospital covers the entire city).

At the second stage, a certain percentage of patients are transferred from the primary institutions to the county hospitals for treatment. Thus, the primary medical institutions become new demand points, and the county hospitals become supply points. The calculation formula of accessibility at this stage is defined as:

$$P_{k1}=\alpha \sum_{k\in (d_{kp}\leq d_{1})}P_{k}$$ (8)

$$R_{c}=\frac{S_{c}}{\sum_{p\in \left(d_{p}{}_{c}\leq d_{1}\right)}P_{k1}}$$ (9)

$$A_{i}{}^{c}=\sum_{p\in \left(d_{ip}\leq d_{0}\right)}\left[\sum_{c\in \left(d_{pc}\leq d_{1}\right)}R_{c}f\left(d_{pc}\right)G_{pc}\right]$$ (10)

where *P*_*k*1_ is the population referred from the primary medical institution to the county hospitals; *α* is the referral rate of the primary medical institution to the county hospitals; *d*_*pc*_ is the distance cost from the primary medical institutions to the county hospitals; *S*_*c*_ is the capacity of the country hospital; *f* (*d*_*pc*_) is the Gravity-type distance decay function from the primary medical institutions to the country hospitals.

The third stage is from primary medical institutions to municipal hospitals. If the patient for treatment is seriously ill, the primary institution can directly refer the patient to the municipal hospitals with a higher medical level. In this way, the patient can receive the most effective treatment in the shortest time. At this stage, the demand point is the primary medical institutions, and the supply point is the municipal hospitals. The calculation formula is expressed as follows:

$$P_{k2}=(\beta +\alpha \gamma )\sum_{k\in (d_{km}\leq d_{2})}P_{k}$$ (11)

$$R_{m1}=\frac{S_{m}}{\sum_{p\in \left(d_{p}{}_{m}\leq d_{2}\right)}P_{k2}}$$ (12)

$$A_{i}^{m1}=\sum_{p\in (d_{ip}\leq d_{0})}\left[\sum_{m\in \left(d_{pm}\leq d_{2}\right)}R_{m1}f\left(d_{pm}\right)G_{pm}\right]$$ (13)

where *P_k_*_2_ is the population from the primary institutions and the country hospitals to municipal hospitals; *β* is the referral rate of the primary medical institution to the municipal hospitals; *γ* is the percent of patients needing to be referred to the municipal hospitals from the country hospitals; *d*_*pm*_ represent the cost of distance from the primary institutions to the municipal hospitals; *S*_*m*_ is the capacity of the municipal hospitals; *f* (*d*_*pm*_) is the Gravity-type distance decay function from the primary medical institutions to the municipal hospitals.

The last stage is from the country hospitals to the municipal hospitals. In addition to the direct referral from the primary institutions, some patients in the municipal hospitals are referred from the county hospitals. The supply and demand sides of this stage are the municipal hospitals and the county hospitals respectively. The formula can be written as:

$$R_{m2}=\frac{S_{m}}{\sum_{c\in \left(d_{c}{}_{m}\leq d_{2}\right)}P_{k2}}$$ (14)

$$A_{i}^{m2}=\sum_{p\in (d_{ip}\leq d_{0})}\left[\sum_{m\in \left(d_{cm}\leq d_{2}\right)}R_{m2}f\left(d_{cm}\right)G_{cm}\right]$$ (15)

### The calculation of the hospital capacity and the referral rates

To study the accessibility of residents to medical resources under the hierarchical diagnosis and treatment system, the key issue is how to determine the referral rate of each referral stage. This paper simulated the size of the population supported by the hospital in different scenarios based on the bed utilization rate. Then the referral rate of hospitals can be obtained. The calculation formula is as follows:

$$C=\frac{By*1000*k}{N}$$ (16)

where *C* is the carrying capacity of hospitals, *N* is the number of beds per thousand. In 2015, the General Office of the State Council of China issued the "National Medical and Health Service System Planning Outline (2015–2020)", which first put forward the quantitative indicators for the scale of medical institutions to develop beds. The "Planning Outline" proposed that by 2020, the number of beds in medical and health institutions will be limited at 6 per 1,000 permanent residents. For the primary medical and health institutions, the number of beds will be 1.2 and the public hospitals will be 3.3. *By* is the bed of hospitals; *k* represents the bearing capacity coefficient of the hospital.


$$\lambda =Median\left\{\frac{C}{p_{k}}\right\}$$ (17)

Where *λ* is the referral rate; *C* is the population carried by various types of hospitals in different situations; *p*_*k*_ is the population in the administrative region.

### Design of scenario

China has been working on the perfection of the hierarchical diagnosis and treatment system to further improve the utilization efficiency and the whole benefit of medical resources. Current aspect of China is that there is a co-existence of the hybrid and the hierarchical diagnosis and treatment. From the perspective of accessibility and considering the carrying capacity of the hospital, this study set three scenarios based on the hierarchical diagnosis and treatment system: business-as-usual scenario, general public health events scenario and major public health events scenario. The convenience of access to medical resources for residents in different situations and the resulting regional differences were analyzed.

#### Business-as-usual scenario

This scenario is an ordinary scenario in which no public health cases have occurred and medical institutions are in normal operation. According to the China Health Statistical Yearbook, the bed occupancy rate in China is generally above 80%. Therefore, in this scenario, the carrying capacity coefficient of the county hospitals and the municipal hospitals is set to 80%. The hierarchical diagnosis and treatment system requires patients must seek medical attention at the primary medical institutions, so the carrying capacity coefficient of the primary medical institutions is set to 100%.

#### General public health case scenario

Hospitals operate at full capacity when there are general public health cases. Thus, the carrying capacity of high-grade hospitals set to 100% in this scenario.

#### Major public health case scenario

When there is a major public health event, the number of people seeking medical treatment can exceed the carrying capacity of the hospital. Due to limited service capacity, the primary medical institutions are mainly responsible for the referral of patients. The treatment of patients is mainly concentrated in the county and the municipal hospitals. In this scenario, the carrying capacity coefficient of the primary medical institutions is set to 100%. The county and the municipal hospitals are overloaded. In order to ensure the normal operation of hospitals, the hospitals need to limit the number of patients strictly. The study set the carrying capacity coefficient of the county and the municipal hospitals to 120%.

## Results

### Results of multi-scenario simulation

#### Referral rates between different hierarchies of hospitals

**Fig 2:**
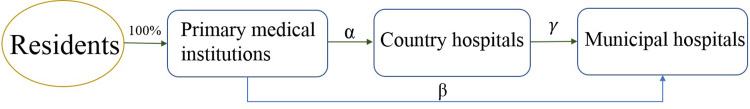
Residents’ treatment process under the hierarchical diagnosis and treatment system.

Residents’ treatment process under the hierarchical diagnosis and treatment system is shown in Fig 2. According to the carrying capacity of hospitals at different levels, this paper simulated the referral rates of hospitals (use Eqs 16 and 17) in different scenarios by setting three scenarios: Business-as-usual scenario, General public health case scenario, and Major public health case scenario. In the Business-as-usual scenario, the referral rate from the primary medical institutions to the county hospitals was 43%, and the referral rate from the county to the municipal hospitals was 63%. When there is a general public health event, the referral rate from the primary medical institutions to the county hospitals is 54%, and the referral rate from the county-level to the municipal hospitals is 67%.

When there is a major public health event, the county and municipal hospitals become the main places for providing medical services. At this time, the referral rate of the primary medical institutions to the county hospitals is 65%, while the referral rate from the county hospitals to municipal hospitals reaches up to 70%. The primary medical institutions can directly refer patients to municipal hospitals according to the patient’s condition. In this way, the referral rate in this scenario is difficult to estimate. Based on relevant literatures, this paper uniformly set the referral rate of the primary medical institutions to municipal hospitals to 10%.

#### The general overview of medical service accessibility in China under three scenarios

The accessibility of medical facilities (calculated by Eq 5) in China shows obvious spatial differences. In summary, the accessibility of the east and central China is much better, while that of southwest and northeast China is poor. But the performance of some areas is different. For example, the accessibility of Fujian Province in the eastern coastal area of China is generally poor, which is inconsistent with the surrounding provinces. In three scenarios, the accessibility of medical facilities in China presents a radial distribution pattern centered on the provincial capital city. Meanwhile, the research unit of this paper is small and leads to a fragmented distribution of medical facilities accessibility in China. Except for provincial capitals and some large cities with rich medical resources, the accessibility of other regions shows a staggered distribution of high and low values.

### Comparison of spatial distribution of the accessibility in three scenarios

#### The spatial distribution characteristics of the medical accessibility in three scenarios

Under different scenarios, the residents’ accessibility to medical services has significant spatial differences, which are shown as follows:

1. The ability of the provincial capital to radiate outward weakened, which is most obvious in Henan and Shaanxi provinces (Fig 3a11-3a13 and 3a21-3a23). In the business-as-usual scenario of the medical and health services, the medical resources of Zhengzhou, the capital of Henan Province, can radiate to four prefecture-level cities (i.e., Kaifeng, Xinxiang, Jiaozuo and Luoyang), and jointly built a ring-shaped high-quality and efficient medical network with Zhengzhou as the core. In the General public health case scenario, the outward radiation effect of high-quality medical resources in Zhengzhou decreased, and there is a decline in the accessibility of medical services in surrounding cities. When there is a major public health event, the outward radiation effect of medical resources in Zhengzhou further reduced, and surrounding cities mainly relied on their own medical networks to provide residents with effective medical services. The Shaanxi Province formed a belt-like high-quality and efficient network system with Xi’an City as the center, as well as Weinan, Xianyang, and Tongchuan City. In the General public health case scenario and the Major public health case scenario, the accessibility of surrounding cities decreased, indicating that the radiation capacity of medical resources in Xi’an gradually weakened.

**Fig 3:**
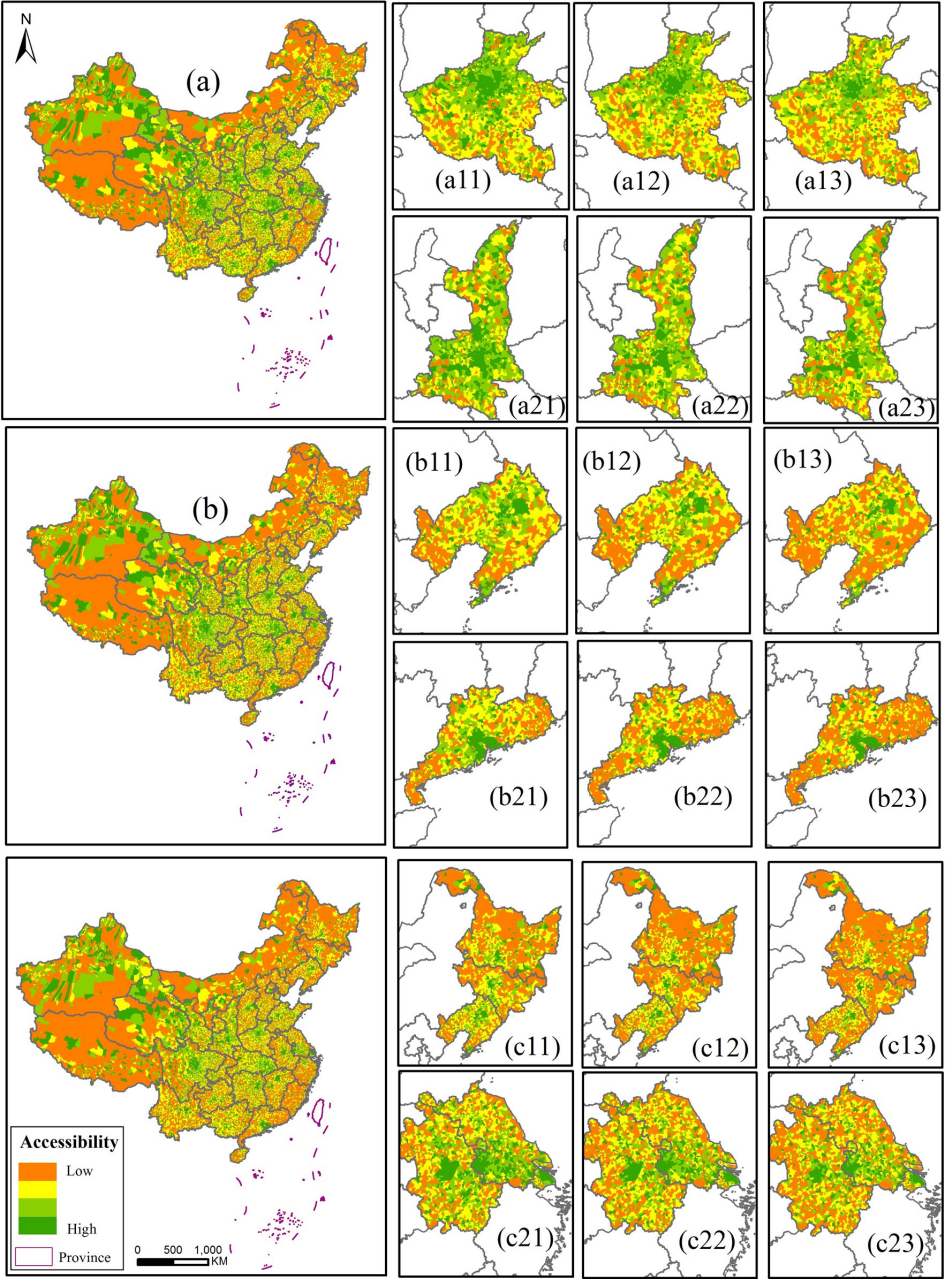
Spatial distribution of residents’ medical accessibility under three scenarios in China. (a) the accessibility under the Business-as-usual scenario; (b) the accessibility under the General public health case scenario;(c) the accessibility under the Major public health case scenario.
2. There is visible disparities within the provinces (Fig 3b11-3b13 and 3b21-3b23). In the business-as-usual scenario, the difference in the accessibility of residents to seek medical care within the provinces was not significant. When presented with the public health events, regional differences in the province became increasingly prominent, especially in Liaoning and Guangdong Province. When it comes to the General public health case, the Liaodong area in Liaoning Province and the coastal areas east of Dalian City, as well as the eastern and western Guangdong Province, had poor access to medical services. Especially occurring the major public events, the medical institutions in the above-mentioned areas cannot provide efficient and high-quality medical and health services for the residents of the jurisdiction. It also reflects the inequity of the accessibility of medical services for residents to varying degrees in different locations of the province.
3. The structure of the high-efficiency medical resource translated from the group corridor to an independent core structure. Due to the short distance between cities and the abundant medical resources, the radiation ability to the surrounding area is strong, and a cross-provincial high-efficiency medical resource corridor structure is formed (Fig 3c11-3c13 and 3c21-3c23). For example, the high-efficiency medical resource corridor of the three northeastern provinces was formed by Shenyang-Changchun-Harbin and the high-efficiency medical resource corridor of the Yangtze River Delta was with Nanjing-Changzhou-Suzhou-Shanghai as the axis. However, in the General public health case scenario and the Major public health case scenario, the referral rate continued to increase, resulting in the sharp increase of the population carried by county and municipal hospitals. And the accessibility of medical treatment in each city gradually declined. At this time, the outward radiation capacity of urban medical facilities weakened leading to the gradual disintegration of the high-efficiency medical resource corridor formed by urban clusters, and transforming into the independent core structure.

#### The impact of the public health event on the accessibility

We analyzed the impact of public health events on accessibility by calculating the mean and standard deviation of accessibility in different scenarios. It can be seen from the Table 3 that the public health events can directly affect the accessibility to medical treatment for residents. Compared with the Business-as-usual scenario, the accessibility value decreased by 11% in the General public health case scenario. The accessibility decreased by 18% in the Major public health case scenario. The three scenarios were established according to different referral rates. It can be found in our study that with the increase of referral rate, the difference in the accessibility of residents to medical treatment gradually decreased, indicating that it is more equal to the residents among different regions for obtaining medical treatment.

**Table 3:** Average and standard deviation of residents’ medical accessibility under three scenarios.

	business-as-usual scenario	General public health case scenario	Major public health case scenario
Mean	0.00433	0.003854	0.003536
Standard deviation	0.006762	0.006114	0.0057

**Fig 4:**
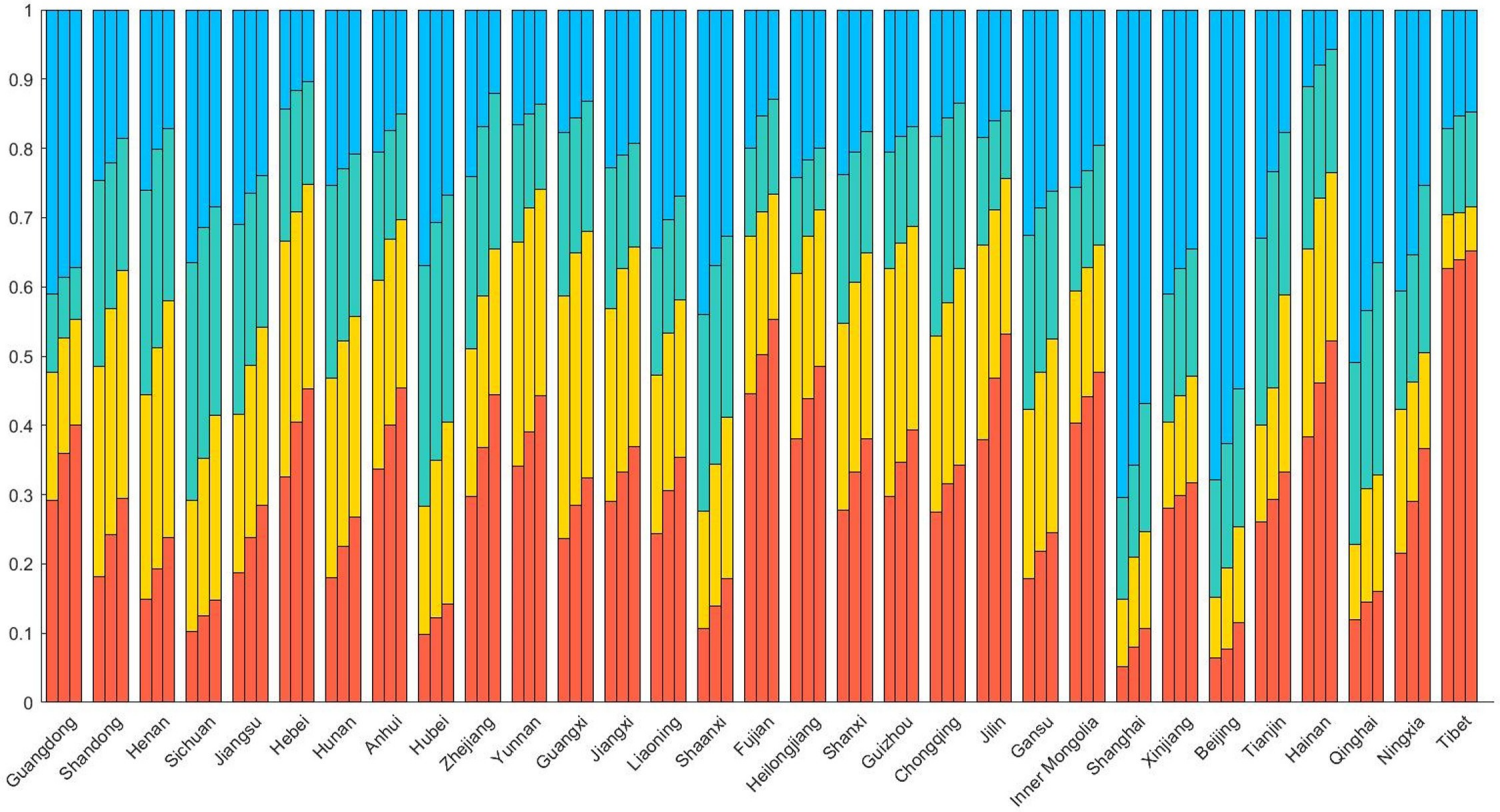
The proportion of population under different accessibility types of three scenarios in each province.

In this paper, the accessibility value was divided into four categories: low, lower, higher, and high. We counted the population of four categories in three scenarios at the provincial scale with the aim to further analyze the impact of public health events on the accessibility of residents to medical treatment (Fig 4). In the three scenarios, the population size of four categories in provinces showed obvious consistency and local differences. The areas with the best accessibility are Beijing and Shanghai in China. More than 85% of the population of Beijing and Shanghai are located in the areas with higher and high accessibility values. The Tibet Autonomous Region has the worst accessibility, with more than 60% of the population in the low-value area of accessibility. With the occurrence of the public health events, the population in low-value area of accessibility increased, among which the proportion of the population in low-value areas such as Ningxia, Jilin and Zhejiang provinces increased by about 15%. The proportion of population in high-value areas showed an overall downward trend. The areas with a larger proportion of decline were located in Ningxia, Qinghai and Tianjin, where the total population was few.

### The contribution rate of medical resources at different levels toward accessibility

**Fig 5:**
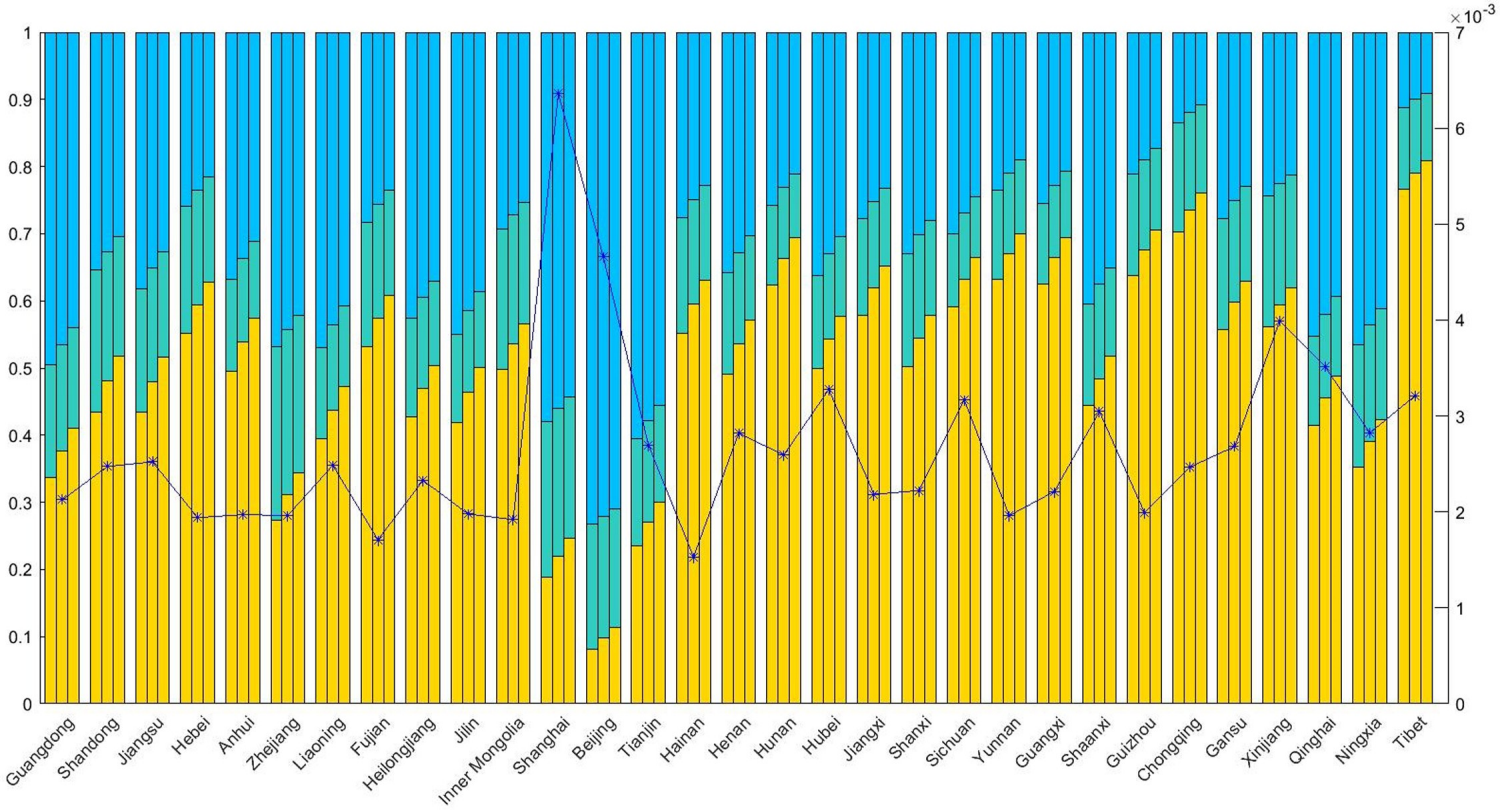
The contribution rate of medical resources at different levels toward accessibility in three scenarios.

As shown in the Fig 5, the contribution rate of medical resources at different levels toward accessibility in three scenarios shows that the primary medical institutions and the municipal hospitals have a larger contribution rate to accessibility, while the contribution rate of the county hospitals is lower. The accessibility contribution rate of the county and municipal hospitals in Shanghai and Beijing is relatively high, indicating that it is more convenient for the residents in this region to obtain high-level medical services. In Tibet, Guizhou, Yunnan, Hunan, Sichuan and Chongqing, which are located in the central and western China, the contribution rate of the primary medical institutions toward accessibility exceeded 60%. It is indicated that primary medical institutions in the above-mentioned region play an important role in providing medical services. In general, the contribution rate of the county hospitals toward accessibility is low. But the service capabilities of the county hospitals in Zhejiang, Shanghai, Shandong and Inner Mongolia Autonomous Region is strong, and the contribution rate of above regions was more than 20%.

## Discussion

This paper used the multi-stage two-step floating catchment area method to study the spatial differences of medical accessibility for residents based on the hierarchical diagnosis and treatment reform. We also constructed three different public health event scenarios to explain the impact of public health events on the accessibility of health care for population.

We modified the two-step floating catchment area method with the aim that the accessibility obtained by the modified method can conform to the medical background of China, and can evaluate the convenience of medical treatment for residents accurately and objectively. First, the reasonable threshold range is the key to calculating accessibility using the two-step moving search method. The travel time threshold in most studies was determined based on the size of the hospital class or the average time from the residential site to the hospital [14, 28, 42, 43]. For China’s health care system, we believe that residents have the equal right to enjoy multi-tier medical resources in the region at the supply level of medical and health services. Therefore, in the first step of the 2SFCA method, we directly divided the total number of beds of hospitals at each level within the threshold range by the total population of hospital service. As for the service areas, the primary medical institutions serve townships, the county hospitals are for the entire county, and the municipal hospitals serve the entire city. Next, in the second stage of the two-step floating catchment area method, previous studies directly summed the supply-demand ratio within the threshold of the demand point after distance decay [39, 44–46]. The effect of distance on accessibility is just considered in this process, but ignoring the differences in the attractiveness of different healthcare facilities to residents. Based on this, we constructed a multi-factor comprehensive selection weight and integrated it into the second stage of accessibility calculation to characterize the difference in accessibility results caused by residents choosing different hospitals. The improved method can overcome the disadvantage that the accessibility is only affected by the distance from the residence to the hospital, and improve the accuracy of accessibility calculation while enriching the application scenarios of accessibility. In general, we believe that the overall distribution of medical resources is fair, and the accessibility of medical care for residents in different areas is affected by their location and choice of hospital.

Our study found significant spatial variation in the accessibility of healthcare facilities in China, with East and Central China having better accessibility overall and Southwest and Northeast China relatively weaker. This is consistent with the findings of existing studies [33, 47]. By comparing the results of the three scenarios of accessibility, we found that the accessibility of medical care for residents of provincial capitals is significantly higher than that of other areas in the province. Especially when there is a public health event, the accessibility of medical treatment in most areas dropped significantly but the provincial capital cities can maintain a high level, indicating that provincial capital cities have better ability to resist risks. In addition, it can be seen that the uneven spatial distribution of medical resources in China [43]. It was noted that as referral rates increased, the equity of access to care for residents continued to increase [36]. Similar findings were found in our study: the equity of residents’ access to care gradually increased when the public health event occurred. By further analyzing the reasons, we found that when a public health event occurs, the lower capacity of primary care institutions can lead to more patients being referred to county and prefectural hospitals for care. In this way, more residents have access to high-grade medical services, which in turn can improve equity of access. In addition, the high referral rate caused by the public health events can lead to the improvement in the fairness of medical treatment, partly because the mismatch between the inverted triangle model of China’s medical resources and the current hierarchical diagnosis and treatment system. By analyzing the contribution of hospitals at each level to accessibility, we found that the primary institutions in Tibet, Guizhou, Yunnan, Hunan, Sichuan and Chongqing in the central and western regions of China have a higher contribution to accessibility. Residents in the above-mentioned areas can easily obtain primary medical and health services, but it is more difficult for them to seek high-level health services by referrals. The situation can be more serious in the context of public health events. Moreover, these areas are located in areas with complex topography in China [48]. In the future, the accessibility to high-grade hospitals for residents should be improved by upgrading transportation infrastructure.

However, our article still has some limitations. First of all, this paper regarded the population of the settlement as a whole without taking into account individual differences, such as gender, age, economic characteristics, etc. Second, when setting the referral rate, this paper viewed the hospitals of the same level as a whole, and divided the hospitals into the primary medical institutions, the county hospitals and the municipal hospitals. The research object is Chinese mainland, which is relatively large. Thus, the differences in referral rates among hospitals at the same level were not considered in our study.

## Conclusion

In the context of the hierarchical diagnosis and treatment system, this paper simulates the referral rate among three-tier medical institutions in different scenarios. At the same time, a multi-factor comprehensive selection weight is proposed to characterize the selection behavior of residents towards the hospital, and integrated into the calculation process of the two-step floating catchment area method, so as to analyze the spatial differences in the accessibility of residents to medical treatment in different scenarios. The conclusions from this study are summarized as follows: With the occurrence of public health events, the referral rate of the high-level hospitals has gradually increased. Especially when there is major public health event, the county and the municipal hospitals are the main places to provide medical services. Based on the hierarchical diagnosis and treatment system, there are obvious spatial differences in the accessibility of residents to seek medical treatment. The eastern and central China are better, while the southwestern and northeastern regions are relatively poor. Having analyzed the contribution rate of medical resources at three-tier to accessibility, it can be seen that the service capacity of the county medical institutions in China is relatively poor. At the same time, the contribution rate of accessibility of the primary institutions in the central and western China is too high, indicating that the service capacity of the high-level medical institutions in these regions is insufficient.
